# Supplementary material for: Sputum scarcity and respiratory sample availability among children with presumptive tuberculosis in high burden countries: a systematic review and meta-analysis
Source: medRxiv. 2025 Nov 4:2025.11.02.25339327. Preprint. [Version 1] doi: 10.1101/2025.11.02.25339327 (PMC12637727; doi:10.1101/2025.11.02.25339327)
Supplement: Supplement 1 [file media-1.pdf]

Sputum scarcity and respiratory sample availability among children with presumptive tuberculosis in high burden countries: a systematic review and meta-analysis

Supplemental Material

Table S1. Search Strategy

**PubMed**	
#1	"Mycobacterium tuberculosis"[MeSH] OR "Tuberculosis"[MeSH] OR Tuberculo*[tiab] OR TB[tiab]
#2	"sputum"[MeSH] OR sputum[tiab] OR "respiratory aspiration"[MeSH] OR "gastric aspirat*"[tiab] OR "gastric lavag*"[tiab] OR "nasopharyngeal aspirat*"[tiab] OR "string test*"[tiab] OR sputa[tiab] OR expectorat*[tiab] OR phlegm*[tiab]
#3	"Urine"[Mesh] OR "Feces"[Mesh] OR "Blood"[Mesh] OR "Serum"[Mesh] OR urin*[tiab] OR stool[tiab] OR fece*[tiab] OR blood[tiab] OR serum*[tiab] OR swab*[tiab]
#4	#2 OR #3
#5	"child"[Mesh] OR "pediatrics"[Mesh] OR childhood*[tiab] OR child*[tiab] OR pediatric*[tiab] OR paediatric*[tiab] OR infan*[tiab]
#7	#1 AND #4 AND #5
#8	"2010/01/01"[PDat] : "2024/6/30"[PDat]

**Embase**	
#1	'Mycobacterium tuberculosis'/exp OR 'tuberculosis'/exp OR Tuberculo*:ti,ab,kw OR TB:ti,ab,kw
#2	'sputum'/exp OR 'sputum examination'/exp OR sputum*:ti,ab,kw OR aspirat*:ti,ab,kw
#3	'urine'/exp OR 'feces'/exp OR 'blood'/exp OR 'serum'/exp OR 'feces analysis'/exp OR 'urinalysis'/exp OR urin*:ti,ab,kw OR stool:ti,ab,kw OR fece*:ti,ab,kw OR blood:ti,ab,kw OR serum*:ti,ab,kw OR swab*:ti,ab,kw
#4	#2 OR #3
#5	child*:ti,ab,kw OR pediatric*:ti,ab,kw OR paediatric*:ti,ab,kw OR infan*:ti,ab,kw
#6	#1 AND #4 AND #5
	Publication date filter: 2010-2024
	Filter: Embase only
	Filter: all categories under 18
	Filter: humans, only English

Table S2. Template for Risk of Bias assessment

**Domain**<br>**Signaling Question**	**Accepted values and answers**
**Domain 1: Patient Selection**<br>Could the selection of patients have introduced bias?	
1. Was a consecutive or random sample of patients enrolled?	Random → yes<br>Consecutive → yes<br>Convenience → no<br>NR → unsure
2. Was a case-control design avoided?	Cohort → yes<br>Cross-sectional → yes<br>Case-control → no<br>NR → unsure
3. Did the study avoid inappropriate exclusions? (e.g. previous TB, HIV, unable to provide an ERS)	Yes → yes<br>No → no<br>NR → unsure
Scoring:<br>Yes on ≥ 2 questions → Low<br>No on ≥ 2 questions → High<br>Unsure on ≥ 2 questions → Unsure	
**Domain 2: Assessment of the Outcome**<br>Is there a concern that the included participants do not match the review question?	
1. Were methods of ERS collection described in sufficient detail?	Yes → yes<br>No → no<br>NR → unsure
2. Were the results of ERS collection available for all, or nearly all, participants?	Yes → yes<br>No → no<br>NR → unsure
3. Was information on sputum quality (e.g. salivary samples) reported?	Yes → yes<br>No → no<br>NR → unsure
Scoring:<br>Yes on ≥ 2 questions → Low<br>No on ≥ 2 questions → High<br>Unsure on ≥ 2 questions → Unsure	

Table S3. Characteristics of included studies

Study ID	Design; DTA	Country (-ies)	Clinical setting	Healthcare level	N pts	TB prevalence (%)	HIV prevalence (%)	Previous TB (%)	SAM prevalence (%)	Collection method	Number samples	Collect time	Proportion scarcity/ without sample	RoB
Ainan 2021 (1)	CSS; Yes	Tanzania	Mixed	Mixed	253	3.6	6.5	NR	NR	ES, GA	2	NR	GA: 0.04	Low, High
Bates 2013 (2)	CSS; Yes	Zambia	Inpatient	Tertiary	1037	6.2	30.5	NR	NR	ES, GA	NR	NR	ES: 0.52<br>GA: 0.00<br>ES<5: 15/663 with sample	Low, Low
Cardoso 2022 (3)	CSS; No	Brazil	Mixed	Tertiary	33	19.0	NR	NR	NR	ST	1	Spot	ST: 0.21	Low, Low
Chibolela 2023 (4)	CSS; No	Zambia	NR	Tertiary	116	11.4	23.7	NR	35.1	GA	1	NR	GA: 0.00	Low, Low
Cox 2022 (5)	CSS; Yes	South Africa	NR	Tertiary	328	30.9	19.6	7.6	29.9	IS w/suction	2	1-2 days	IS: 0.02	Low, High
Dayal 2021 (6)	CSS; Yes	India	NR	Tertiary	114	51.7	NR	NR	10.5	IS, GA	1	Spot	GA: 0.00	Low, High
Dubale 2022 (7)	CSS; Yes	Ethiopia	NR	Tertiary	152	6.7	NR	NR	35.5	ES, GA	1	NR	GA: 0.00	Low, High
Ebonyi 2020 (8)	CSS; Yes	Nigeria	Outpatient	Tertiary	103	45.6	NR	NR	68.7	ES, GA	1	Spot	ES: 0.29<br>GA: 0.00<br>ES<5: 4/58 with sample	Low, High
Hanrahan 2019 (9)	Cohort; No	South Africa	Outpatient	Primary	119	3.0	18.0	4.0	16.0	ES, NPA, IS, GA	1 ES, 2 NPA, 1 IS, 1 GA	Spot	ES: 0.41<br>IS: 0.02<br>GA: 0.23<br>NPA: 0.04<br>ES<5: 4/102 with sample	Low, Low
Hasan 2017 (10)	CSS; Yes	Pakistan	NR	Tertiary	50	18.0	NR	NR	NR	ES, GA	1	NR	ES: 0.69<br>GA: 0.00	Low, Low
Jaganath 2021 (11)	CSS; Yes	Uganda	Mixed	Tertiary	217	10.8	13.0	1.4	NR	ES, IS, NPA, GA	2	NR	ES: 0.54	Low, High
Kabir 2018 (12)	CSS; Yes	Bangladesh	Inpatient	Tertiary	102	15.7	NR	NR	86.2	ES, GA	1	Spot	ES: 0.64<br>GA: 0.00	Low, Low
Kabir 2021 (13)	CSS; Yes	Bangladesh	Inpatient	Tertiary	447	16.1	NR	2.5	41.8	IS w/ suction	1	NR	IS: 0.00	Low, Low
KasaTom 2018 (14)	CSS; Yes	Papua New Guinea	Inpatient	Tertiary	100	28.0	18.3	NR	48.4	ES, GA	2 ES, 1 GA	Spot	ES: 0.15<br>GA: 0.05<br>ES<5: 3/60 with sample	Low, Low

Khambati 2024 (15)	CSS; No	Kenya	Mixed	Mixed	300	10.5	24.3	NR	NR	GA, ST	2 each	3 days	GA: 0.02<br>ST: 0.03	Low, Low
Kroidl 2015 (16)	CSS; Yes	Tanzania	Outpatient	Tertiary	180	13.6	51.0	NR	NR	IS	3	NR	IS: 0.01	Low, Low
LaCourse 2018 (17)	RCT; Yes	Kenya	Inpatient	Tertiary	181	7.9	100	NR	48.1	IS, GA	2	1-2 days	GA: 0.04	Low, Low
Menon 2011 (18)	CSS; No	India	NR	Tertiary	52	36.5	0	NR	NR	GA	Up to 3	NR	GA: 0.00	Low, Low
Moore 2011 (19)	CSS; No	South Africa	NR	Primary	270	10.7	18.0	NR	NR	IS w/suction	2	NR	IS: 0.01*	Low, Low
Moore 2017 (20)	CSS; No	South Africa	Inpatient	Tertiary	920	2.9	12.5	NR	15.9	IS, GA	2	1-2 days	IS: 0.09<br>GA: 0.02	Low, Low
Mutabazi 2020 (21)	CSS; No	Tanzania	Mixed	Mixed	263	5.2	100	9.5	4.4	ES, GA	2 ES, 1 GA	1-2 days	ES: 0.15<br>GA: 0.07	Low, Low
Myo 2018 (22)	CSS; Yes	Myanmar	NR	Tertiary	231	16.5	19.0	13.0	NR	GA	1	NR	GA: 0.00	Low, Low
Nansumba 2016 (23)	CSS; No	Uganda	Mixed	Tertiary	137	10.2	31.0	NR	3.6	ST, IS w/ suction	2 each	2 days	IS: 0.08<br>ST: 0.16	Low, Low
Nicol 2019 (24)	CSS; Yes	South Africa	NR	Tertiary	165	24.2	10.9	NR	NR	IS w/suction	2	NR	IS: 0.00	Low, Low
Orikiriza 2018 (25)	Cohort; Yes	Uganda	Mixed	Tertiary	385	4.3	31.2	2.0	19.0	ES, IS w/suction	2	2 days	ES: 0.36<br>IS: 0.11	Low, High
Orikiriza 2022 (26)	CSS; Yes	Uganda	Inpatient	Tertiary	213	5.5	32.9	NR	84.0	ES, IS w/ suction, NPA, GA	2	2 days	ES: 0.22<br>NPA: 0.03<br>GA: 0.03<br>ES<5: 5/39 with sample	Low, High
Pang 2014 (27)	CSS; Yes	China	NR	Tertiary	211	8.1	NR	NR	NR	GA	1	Spot	GA: 0.00	Low, Low
Planting 2014 (28)	CSS; No	South Africa	Inpatient	Tertiary	843	18.6	23.8	10.4	15.6	IS w/ suction	2	1-2 days	IS: 0.02*	Low, Low
Sabi 2016 (29)	CSS; No	Tanzania	Mixed	Tertiary	192	5.2	15.1	NR	49.5	IS w/ suction	1	NR	IS: 0.03	Low, Low
Sekadde 2013 (30)	CSS; Yes	Uganda	Mixed	Tertiary	255	14.0	41.6	7.2	27.2	IS	1	Spot	IS: 0.02	Low, Low
Singh 2021 (31)	CSS; Yes	India	Outpatient	Tertiary	356	27.4	NR	NR	NR	IS, GA	2 each	2 days	IS: 0.06<br>GA: 0.00	Low, High
Singh 2023 (32)	CSS; No	India	Inpatient	NR	4356	2.0	0	NR	100	GA	1	NR	GA: 0.02	Low, Low
Sreedeep 2020 (33)	CSS; Yes	India	NR	Tertiary	55	13.3	11.7	NR	35.0	ES, GA	2	2 days	GA: 0.00	Low, Low
Tiwari 2015 (34)	CSS; Yes	India	NR	NR	100	36.0	NR	NR	NR	ES, GA	2 ES, 3 GA	2-3 days	ES: 0.08<br>GA: 0.00	Low, High
Yalamanchi 2023 (35)	CSS; No	India	Inpatient	Tertiary	255	10.2	NR	NR	NR	IS, GA	2 each	2 days	IS: 0.02<br>GA: 0.00	Unsure High

Yenew 2024 (36)	CSS; Yes	Ethiopia	Mixed	Mixed	896	7.6	13.2	NR	NR	ES, GA	1	Spot	GA: 0.00	Low, Low
Studies reporting results of suctioning if unable to cough after nebulization:<br>Moore 2011: Of the 496 IS procedures, 296 (60%) samples were obtained by coughing and 200 (40%) by suctioning.<br>Planting 2014: Of the 1257 IS procedures, 264 (21.0%) were obtained by coughing and 993 (79.0%) by suctioning.														

Legend: (abbreviations)
Design: CSS=cross-sectional study; RCT=randomized controlled trial
DTA=diagnostic test accuracy (yes, no)
Clinical setting: inpatient, outpatient, mixed inpatient and outpatient, not reported (NR)
Healthcare level: Primary, Secondary, Tertiary, mixed levels
N pts: number of participants attempting respiratory sample collection
SAM = severe acute malnutrition
Collection method: self-expectorated sputum (ES), induced sputum (IS), with suctioning if child unable to cough (w/suction), gastric aspirate (GA), nasopharyngeal aspirate (NPA), self-expectorated sputum in children <5 years (ES<5)
Number of samples attempted: 1-2, more than 2, not reported (NR)
Time of sample collection: spot, 1-2 days, 3 days or more, not reported (NR)
Risk of Bias (RoB): Patient selection, Applicability

Figure S1. Meta-analysis of proportion children under 5 years able to provide self-expectorated sputum

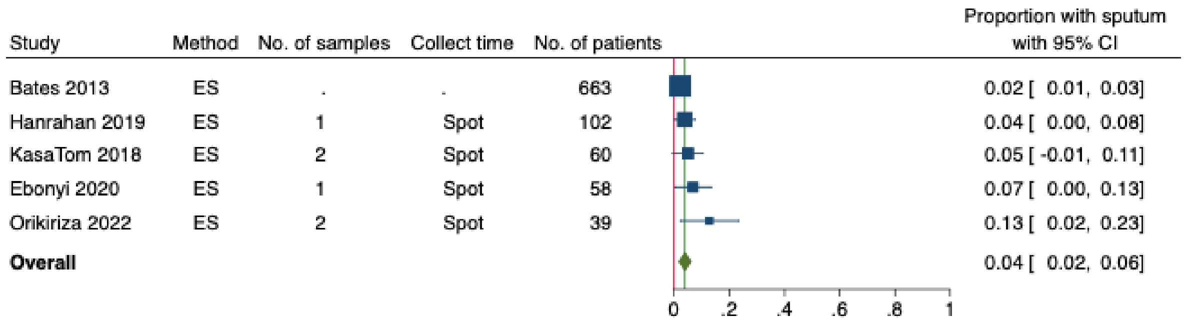

Figure S2. Meta-analysis of proportion sputum scarcity for collection of self-expectorated sputum in children 5-15 years

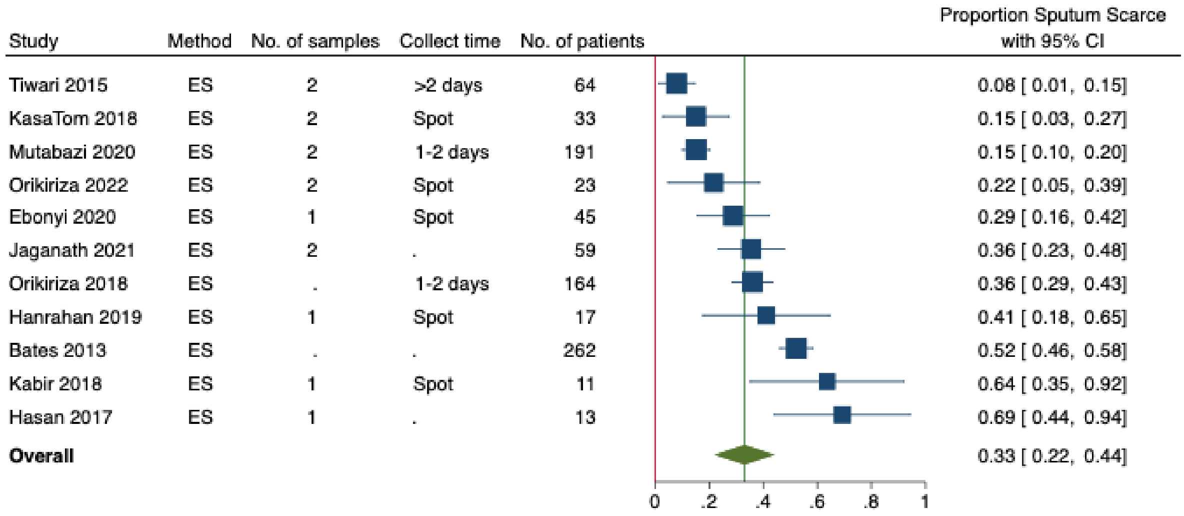

Figure S3. Meta-analysis of proportion sputum scarcity for collection of 1-2 self-expectorated spot samples in children 5-15 years

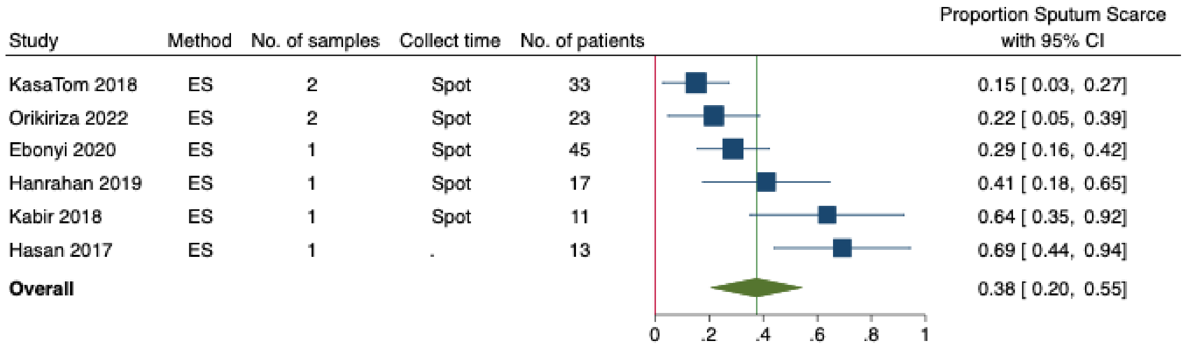

Figure S4. Meta-analysis of proportion sputum scarcity in studies with mixed HIV population for collection of self-expectorated sputum in children 5-15 years

Study	Method	No. of samples	Collect time	%CLHIV	No. of patients	Proportion Sputum Scarce with 95% CI
KasaTom 2018	ES	2	Spot	18.3	33	0.15 [0.03, 0.27]
Mutabazi 2020	ES	2	1-2 days	100.0	191	0.15 [0.10, 0.20]
Orikiriza 2022	ES	2	Spot	32.9	23	0.22 [0.05, 0.39]
Jaganath 2021	ES	2	.	13.0	59	0.36 [0.23, 0.48]
Orikiriza 2018	ES	.	1-2 days	31.2	164	0.36 [0.29, 0.43]
Hanrahan 2019	ES	1	Spot	18.0	17	0.41 [0.18, 0.65]
Bates 2013	ES	.	.	30.5	262	0.52 [0.46, 0.58]
**Overall**						0.31 [0.20, 0.42]

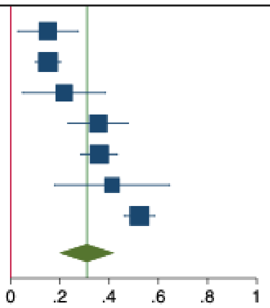

Figure S5. Meta-analysis of proportion sputum scarcity in studies with mixed SAM population for collection of self-expectorated sputum in children 5-15 years

Study	Method	No. of samples	Collect time	%SAM	No. of patients	Proportion Sputum Scarce with 95% CI
KasaTom 2018	ES	2	Spot	48.4	33	0.15 [0.03, 0.27]
Orikiriza 2022	ES	2	Spot	84.0	23	0.22 [0.05, 0.39]
Ebonyi 2020	ES	1	Spot	68.7	45	0.29 [0.16, 0.42]
Hanrahan 2019	ES	1	Spot	16.0	17	0.41 [0.18, 0.65]
Kabir 2018	ES	1	Spot	86.2	11	0.64 [0.35, 0.92]
**Overall**						0.31 [0.17, 0.45]

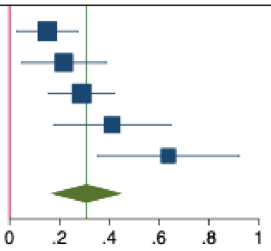

Figure S6. Meta-analysis of proportion sputum scarcity for collection of induced sputum, children<15 years

Study	Method	No. of samples	Collect time	Suction	No. of patients	Proportion Sputum Scarce with 95% CI
Kabir 2021	IS	1	.	Yes	447	0.00 [-0.00, 0.00]
Nicol 2019	IS	2	.	Yes	165	0.00 [-0.00, 0.00]
Kroidl 2015	IS	up to 3	.	NR	180	0.01 [-0.01, 0.02]
Moore 2011	IS	2	.	Yes	270	0.01 [-0.00, 0.02]
Cox 2022	IS	2	1-2 days	Yes	328	0.02 [0.00, 0.04]
Hanrahan 2019	IS	1	Spot	NR	105	0.02 [-0.01, 0.05]
Planting 2014	IS	2	1-2 days	Yes	843	0.02 [0.01, 0.03]
Sekadde 2013	IS	1	Spot	NR	255	0.02 [0.00, 0.04]
Yalamanchi 2023	IS	2	1-2 days	NR	255	0.02 [0.00, 0.04]
Sabi 2016	IS	1	.	Yes	189	0.03 [0.01, 0.05]
Singh 2021	IS	1	1-2 days	NR	356	0.06 [0.03, 0.09]
Nansumba 2016	IS	2	1-2 days	Yes	137	0.08 [0.03, 0.13]
Moore 2017	IS	2	1-2 days	NR	920	0.09 [0.07, 0.11]
Orikiriza 2018	IS		1-2 days	Yes	284	0.11 [0.08, 0.15]
**Overall**						0.03 [0.01, 0.05]

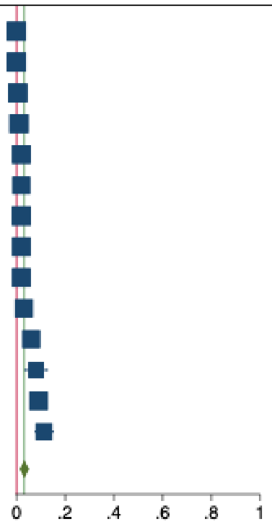

Figure S7. Meta-analysis of proportion sputum scarcity for collection of induced sputum using suctioning if children are unable to cough, children<15 years

Study	Method	No. of samples	Collect time	Suction	No. of patients	Proportion Sputum Scarce with 95% CI
Kabir 2021	IS	1	.	Yes	447	0.00 [-0.00, 0.00]
Nicol 2019	IS	2	.	Yes	165	0.00 [-0.00, 0.00]
Moore 2011	IS	2	.	Yes	270	0.01 [-0.00, 0.02]
Cox 2022	IS	2	1-2 days	Yes	328	0.02 [0.00, 0.04]
Planting 2014	IS	2	1-2 days	Yes	843	0.02 [0.01, 0.03]
Sabi 2016	IS	1	.	Yes	189	0.03 [0.01, 0.05]
Nansumba 2016	IS	2	1-2 days	Yes	137	0.08 [0.03, 0.13]
Orikiriza 2018	IS		1-2 days	Yes	284	0.11 [0.08, 0.15]
**Overall**						0.03 [0.00, 0.06]

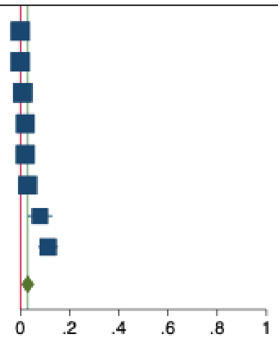

References

1. Ainan S, Furia FF, Mhimbira F, Mnyambwa NP, Mgina N, Zumla A, et al. Xpert® MTB/RIF assay testing on stool for the diagnosis of paediatric pulmonary TB in Tanzania. Public Health Action. 2021;11(2):75-9.
2. Bates M, O'Grady J, Maeurer M, Tembo J, Chilukutu L, Chabala C, et al. Assessment of the Xpert MTB/RIF assay for diagnosis of tuberculosis with gastric lavage aspirates in children in sub-Saharan Africa: a prospective descriptive study. Lancet Infect Dis. 2013;13(1):36-42.
3. Cardoso CAA, Rossoni AMO, Rezende JM, Aurilio RB, Santos R, Ferrarini MAG, et al. String test: a potentially useful tool in the diagnosis of pulmonary tuberculosis in Brazilian children and adolescents. Rev Inst Med Trop Sao Paulo. 2022;64:e27.
4. Chibolela M, de Haas P, Klinkenberg E, Kosloff B, Chunda-Liyoka C, Lungu P, et al. Use of stool swabs in molecular transport media increases access to Xpert Ultra testing for TB in children. Int J Tuberc Lung Dis. 2023;27(8):612-8.
5. Cox H, Workman L, Bateman L, Franckling-Smith Z, Prins M, Luiz J, et al. Oral Swab Specimens Tested With Xpert MTB/RIF Ultra Assay for Diagnosis of Pulmonary Tuberculosis in Children: A Diagnostic Accuracy Study. Clin Infect Dis. 2022;75(12):2145-52.
6. Dayal R, Yadav A, Agarwal D, Kumar M, Kamal R, Singh D, et al. Comparison of Diagnostic Yield of Tuberculosis Loop-Mediated Isothermal Amplification Assay With Cartridge-Based Nucleic Acid Amplification Test, Acid-Fast Bacilli Microscopy, and Mycobacteria Growth Indicator Tube Culture in Children With Pulmonary Tuber. JOURNAL OF THE PEDIATRIC INFECTIOUS DISEASES SOCIETY. 2021;10(2):83-7.
7. Dubale M, Tadesse M, Berhane M, Mekonnen M, Abebe G. Stool-based Xpert MTB/RIF assay for the diagnosis of pulmonary tuberculosis in children at a teaching and referral hospital in Southwest Ethiopia. PLoS One. 2022;17(5):e0267661.
8. Ebonyi AO, Oguche S, Abok, II, Isa YO, Ani CC, Akhiwu HO, et al. Improving the diagnosis of pulmonary tuberculosis using line probe assay and determining the factors associated with the disease in children in Jos, Nigeria. Germs. 2020;10(4):328-37.
9. Hanrahan CF, Dansey H, Mutunga L, France H, Omar SV, Ismail N, et al. Diagnostic strategies for childhood tuberculosis in the context of primary care in a high burden setting: the value of alternative sampling methods. Paediatr Int Child Health. 2019;39(2):88-94.
10. Hasan Z, Shakoor S, Arif F, Mehnaz A, Akber A, Haider M, et al. Evaluation of Xpert MTB/RIF testing for rapid diagnosis of childhood pulmonary tuberculosis in children by Xpert MTB/RIF testing of stool samples in a low resource setting. BMC Res Notes. 2017;10(1):473.
11. Jaganath D, Wambi P, Reza TF, Nakafeero J, Aben EO, Kiconco E, et al. A Prospective Evaluation of Xpert MTB/RIF Ultra for Childhood Pulmonary Tuberculosis in Uganda. J Pediatric Infect Dis Soc. 2021;10(5):586-92.
12. Kabir S, Uddin MKM, Chisti MJ, Fannana T, Haque ME, Uddin MR, et al. Role of PCR method using IS6110 primer in detecting Mycobacterium tuberculosis among the clinically diagnosed childhood tuberculosis patients at an urban hospital in Dhaka, Bangladesh. Int J Infect Dis. 2018;68:108-14.
13. Kabir S, Rahman SMM, Ahmed S, Islam MS, Banu RS, Shewade HD, et al. Xpert Ultra Assay on Stool to Diagnose Pulmonary Tuberculosis in Children. Clin Infect Dis. 2021;73(2):226-34.
14. Kasa Tom S, Welch H, Kilalang C, Tefuarani N, Vince J, Lavu E, et al. Evaluation of Xpert MTB/RIF assay in children with presumed pulmonary tuberculosis in Papua New Guinea. Paediatr Int Child Health. 2018;38(2):97-105.

15. Khambati N, Song R, Smith JP, Bijker EM, McCarthy K, Click ES, et al. Feasibility and utility of a combined nasogastric-tube-and-string-test device for bacteriologic confirmation of pulmonary tuberculosis in young children. Diagn Microbiol Infect Dis. 2024;109(3):116302.
16. Kroidl I, Clowes P, Reither K, Mtafya B, Rojas-Ponce G, Ntinginya EN, et al. Performance of urine lipoarabinomannan assays for paediatric tuberculosis in Tanzania. Eur Respir J. 2015;46(3):761-70.
17. LaCourse SM, Pavlinac PB, Cranmer LM, Njuguna IN, Mugo C, Gatimu J, et al. Stool Xpert MTB/RIF and urine lipoarabinomannan for the diagnosis of tuberculosis in hospitalized HIV-infected children. Aids. 2018;32(1):69-78.
18. Menon PR, Lodha R, Singh U, Kabra SK. A prospective assessment of the role of bronchoscopy and bronchoalveolar lavage in evaluation of children with pulmonary tuberculosis. J Trop Pediatr. 2011;57(5):363-7.
19. Moore HA, Apolles P, de Villiers PJ, Zar HJ. Sputum induction for microbiological diagnosis of childhood pulmonary tuberculosis in a community setting. Int J Tuberc Lung Dis. 2011;15(9):1185-90, i.
20. Moore DP, Higdon MM, Hammitt LL, Prosperi C, DeLuca AN, Da Silva P, et al. The Incremental Value of Repeated Induced Sputum and Gastric Aspirate Samples for the Diagnosis of Pulmonary Tuberculosis in Young Children With Acute Community-Acquired Pneumonia. Clin Infect Dis. 2017;64(suppl_3):S309-s16.
21. Mutabazi SA, Jumanne S, Mpondo BC, Mnzava DP. Prevalence of culture positive Tuberculosis and utility of a clinical diagnostic tool for the diagnosis of Tuberculosis among HIV Infected Children attending HIV/AIDS Care and Treatment in Dodoma Municipality, Central Tanzania. Int J Infect Dis. 2020;96:593-9.
22. Myo K, Zaw M, Swe TL, Kyaw YY, Thwin T, Myo TT, et al. Evaluation of Xpert MTB/RIF assay as a diagnostic test for pulmonary tuberculosis in children in Myanmar. The International Journal of Tuberculosis and Lung Disease. 2018;22(9):1051-5.
23. Nansumba M, Kumbakumba E, Orikiriza P, Muller Y, Nackers F, Debeaudrap P, et al. Detection Yield and Tolerability of String Test for Diagnosis of Childhood Intrathoracic Tuberculosis. Pediatr Infect Dis J. 2016;35(2):146-51.
24. Nicol MP, Wood RC, Workman L, Prins M, Whitman C, Ghebrekristos Y, et al. Microbiological diagnosis of pulmonary tuberculosis in children by oral swab polymerase chain reaction. Sci Rep. 2019;9(1):10789.
25. Orikiriza P, Nansumba M, Nyehangane D, Bastard M, Mugisha IT, Nansera D, et al. Xpert MTB/RIF diagnosis of childhood tuberculosis from sputum and stool samples in a high TB-HIV-prevalent setting. Eur J Clin Microbiol Infect Dis. 2018;37(8):1465-73.
26. Orikiriza P, Smith J, Ssekyanzi B, Nyehangane D, Mugisha Taremwa I, Turyashemererwa E, et al. Tuberculosis diagnostic accuracy of stool Xpert MTB/RIF and urine AlereLAM in vulnerable children. Eur Respir J. 2022;59(1).
27. Pang Y, Wang Y, Zhao S, Liu J, Zhao Y, Li H. Evaluation of the Xpert MTB/RIF assay in gastric lavage aspirates for diagnosis of smear-negative childhood pulmonary tuberculosis. Pediatr Infect Dis J. 2014;33(10):1047-51.
28. Planting NS, Visser GL, Nicol MP, Workman L, Isaacs W, Zar HJ. Safety and efficacy of induced sputum in young children hospitalised with suspected pulmonary tuberculosis. Int J Tuberc Lung Dis. 2014;18(1):8-12.
29. Sabi I, Kabyemera R, Mshana SE, Kidenya BR, Kasanga G, Gerwing-Adima LE, et al. Pulmonary TB bacteriologically confirmed by induced sputum among children at Bugando Medical Centre, Tanzania. Int J Tuberc Lung Dis. 2016;20(2):228-34.

30. Sekadde MP, Wobudeya E, Joloba ML, Ssengooba W, Kisembo H, Bakeera-Kitaka S, et al. Evaluation of the Xpert MTB/RIF test for the diagnosis of childhood pulmonary tuberculosis in Uganda: a cross-sectional diagnostic study. BMC Infect Dis. 2013;13:133.
31. Singh UB, Verma Y, Jain R, Mukherjee A, Gautam H, Lodha R, et al. Childhood Intra-Thoracic Tuberculosis Clinical Presentation Determines Yield of Laboratory Diagnostic Assays. Front Pediatr. 2021;9:667726.
32. Singh M, Dhingra B, Bishnu B, Pandey D, Anand PK, Gupta S, et al. Pulmonary Tuberculosis in Severely Malnourished Children Admitted to Nutrition Rehabilitation Centers: A Multicenter Study. Indian J Pediatr. 2023.
33. Sreedeep KS, Sethi S, Yadav R, Vaidya PC, Angurana SK, Saini A, et al. Loop-mediated isothermal amplification (LAMP) in the respiratory specimens for the diagnosis of pediatric pulmonary tuberculosis: A pilot study. J Infect Chemother. 2020;26(8):823-30.
34. Tiwari S, Nataraj G, Kanade S, Mehta P. Diagnosis of pediatric pulmonary tuberculosis with special reference to polymerase chain reaction based nucleic acid amplification test. Int J Mycobacteriol. 2015;4(1):48-53.
35. Yalamanchi PR, Varma KK. Pulmonary tuberculosis in children: the role of induced sputum in diagnosing the disease. Journal of Cardiovascular Disease Research. 2023;14(4):1263-71.
36. Yenew B, De Haas P, Babo Y, Diriba G, Sherefdin B, Bedru A, et al. Diagnostic accuracy, feasibility and acceptability of stool-based testing for childhood tuberculosis. ERJ Open Research. 2024;10(3):00710-2023.
